# The Evolution of Invertebrate Animals

**DOI:** 10.3390/genes13030454

**Published:** 2022-03-02

**Authors:** Stephanie Bertrand, Hector Escriva

**Affiliations:** Sorbonne Université, CNRS, Biologie Intégrative des Organismes Marins, BIOM, Observatoire Océanologique, F-66650 Banyuls-sur-Mer, France

The current diversity of metazoans has been achieved through a long process of evolution since the appearance of their unicellular ancestor about 1000 Mya. This evolutionary process has generated about 35–37 extant phyla of animals, which are composed by invertebrate animals, with the exception of a single subphylum, the vertebrates. Currently, the number of described living metazoan species is around 1,162,000, among which only about 50,000 are vertebrates (about 5%). In addition, invertebrate animals have been able to adapt to all types of ecosystems, both aquatic and terrestrial, making the study of the diversity and evolution of invertebrates essential to the understanding extant animal biology.

To summarize the history of research on invertebrates or based on invertebrate animals would be too extensive. However, it should be noted that since its creation, the Nobel Prize has been awarded on many occasions to researchers using invertebrate models. Some examples include research using *Drosophila* as a model (e.g., the role of chromosomes in heredity, circadian rhythm, mechanisms of innate immunity, odorant receptors, genetic control of early embryonic development), *Caenorhabditis elegans* (the mechanisms of programmed cell death, RNA interference), sea urchin (key regulators of the cell cycle), sea slug (signal transduction in the nervous system), bees (organization of social and behaviour patterns), crabs (physiological and chemical visual processes), octopus (the ionic mechanisms involved in excitation and inhibition in the peripheral and central portions of the nerve cell membrane), or jellyfish (for the discovery and development of the green fluorescent protein, GFP).

In addition to this long history of invertebrate model-based research, we are now living in an exceptional era for two major reasons: first, because since the first complete genome of an invertebrate animal was sequenced (that of *C. elegans* in 2000), we now have access to about 1000 complete genome sequences of invertebrate animal species (deposited in the NCBI database) and, second, because thanks to the development of simple genome modification techniques, such as CRISPR/Cas9 or TALEN, we can carry out a series of functional experiments that were unthinkable even just a few years ago.

Considering all this, we are pleased to present in this volume entitled “The evolution of invertebrate animals”, new and fascinating studies on different invertebrate lineages focused on several aspects of their biology. This volume contains eight original research articles and three reviews whose focus, ideas, and hypotheses reflect the current diversity and future directions of research using invertebrates as model organisms. This volume is clearly not intended to be an exhaustive collection of studies on invertebrate animals, but we hope that the collection of articles presented here will give a general idea of both the type of studies carried out with invertebrate metazoans and the diversity of animal models used. Thus, we can read works carried out with *Acropora* corals [1], with several mollusc species such as the cephalopod *Nautilus pompilius* [2], the gastropod *Crepidula fornicata* [3] or the bivalve *Mytilus galloprovincialis* [4], as well as a study with the planarian *Schmidtea mediterranea* [5], or with several chordates such as two cephalochordate species (*Branchiostoma lanceolatum* [6] and *Branchiostoma floridae* [7]) and two urochordate species (*Ciona robusta* [8] and *Phallusia mammillata* [4]).

The easier access to transcriptomic and genomic data from non-classical animal models makes the study of the evolution of gene families more comprehensive today. Thus, this volume presents two articles concerning the evolution of gene families. The first one, by Kashimoto and collaborators [1], focuses on the evolution of the fluorescent proteins (FP) gene family, showing an important expansion of FP genes in the genus *Acropora* that has probably contributed to the present-day richness of this coral genus. The second one, by Miglioli and collaborators [9], reviews the present knowledge on the evolution of the nuclear receptor gene family and their functions in marine invertebrates. In addition to the study of the evolution of gene families, access to high throughput data also allows the identification and study of genes involved in biological processes. Thus, the work presented by Setiamarga and collaborators [2] analyses the content of genes encoding shell matrix proteins in the mollusc *Nautilus*, an example of a cephalopod that has not lost its external biomineralized shell. The results demonstrate the presence of such genes in the ancestor of shelled mollusks (Conchiferans) but show that their function in shell formation was acquired during the evolution of this lineage independently in each clade.

Invertebrate models are also extremely useful for studying different biological functions, whether they are conserved among different phyla or specific to an evolutionary lineage. In this sense, Truchado-Garcia and collaborators analysed the role of nitric oxide (NO) in the mollusc *C. fornicata* [3] through the study of the expression of the nitric oxide synthase (*NOS*) gene using whole mount *in situ* hybridization in embryos and larva. This work suggests an involvement of the NO signal in shell formation and neural specification. Another paper in this volume, presented by Pascual-Carreras and colleagues [5], identifies the Wnt/β catenin receptors that specify cell fate and induce the proliferative response during regeneration in the planarian *S. mediterranea*. These data allow a better understanding of the mechanisms through which the “organizers” function during the regeneration process. Finally, Zawisa-Alvarez and collaborators [6] studied the expression of the ADAR (adenosine deaminase acting on RNA) family genes and their putative function in RNA editing in amphioxus. Moreover, this study also presents a new method to identify RNA editing events in polymorphic genomes.

Two other articles in this volume are focused on two biological processes of great importance, the mechanisms at work in synapses and the meiotic division, using different species of tunicate as models. In the first one, Coppola and collaborators [8] studied the expression of the Rab3 interacting molecule-binding protein (*Rimbp*) gene and its expression regulation through the identification of the genetic regulatory elements present in its genomic locus. This work highlights different expression domains for each *Rimbp* isoform in the nervous system of *C. robusta* larvae and will certainly help to understand the function of this family of genes in the context of ascidian embryogenesis. In the second article, McDougall and collaborators [4] studied the process of polar body formation during the meiosis process in *P. mammillata*. The results obtained show that the first polar body becomes tethered to the fertilized egg *via* the second polar body, indicating that the site of first polar body cytokinesis precisely directs the site of the second polar body emission. This process is studied in detail, showing how powerful the use of invertebrate animal models can be in providing an in-depth understanding of general biological processes.

However, before such in-depth studies can be performed, it is first necessary to develop sufficient technical approaches in these unconventional animal models. In this sense, Su and collaborators [7] present here a protocol developed in amphioxus (*Branchiostoma floridae*) for genome editing using the CRISPR/Cas9 technique, which allows obtaining clear KO phenotypes in the F0 of this interesting animal model.

Finally, two review articles complete this volume. The first one, by Hudson and Yasuo [10], focuses on the formation of the chordate central nervous system. Although it is usually thought that in vertebrates it derives from ectoderm cells, the posterior central nervous system (CNS) develops from cells that are neuromesodermal progenitors and that can hence also develop into mesodermal structures. In ascidians, most of the CNS derives from precursors that also give rise to mesodermal tissues, and the authors review here the current knowledge on this subject and on the link between antero-posterior patterning and neural fate acquisition. The second review, by Picard and collaborators [11], presents the different modes of reproduction and sex determination systems in invertebrates, highlighting the impressive collection of possibilities in these animals and, most importantly, the ease and evolutionary speed at which changes between different types of sex determination occur in close evolutionary lineages.

In conclusion, we hope that this modest collection of articles, presenting different aspects of invertebrate animal model research, will convince the readers of the importance of developing experimental techniques and approaches allowing a better understanding of animal biodiversity and the biological functions that such diversity harbours.
